# Clinicopathological and prognostic study of IgA-dominant postinfectious glomerulonephritis

**DOI:** 10.1186/s12882-021-02462-3

**Published:** 2021-07-05

**Authors:** Ziyuan Huang, Bo Chen, Ying Zhou, Yan Liang, Wenxian Qiu, Yinqiu Lv, Xiaokai Ding, Ji Zhang, Chaosheng Chen

**Affiliations:** 1 Department of Nephrology, The First Affiliated Hospital of Wenzhou Medical University, Nanbaixiang, Ouhai District, Zhejiang 325000 Wenzhou, People’s Republic of China; 2 Institute of Chronic Kidney Disease, Wenzhou Medical University, Zhejiang 325000 Wenzhou, People’s Republic of China

**Keywords:** Kidney disease, Clincopathology, Prognosis, Glomerulonephritis, IgA-dominant postinfectious glomerulonephritis

## Abstract

**Background:**

The clinicopathological and prognostic features of IgA-dominant postinfectious glomerulonephritis and its difference from the primary IgA nephropathy remains to be investigated.

**Methods:**

The clinical and pathological data of 6542 patients who underwent renal biopsy from 2009 to 2020 in our hospital were reviewed and 50 patients who met the selection criteria of IgA-dominant postinfectious glomerulonephritis were enrolled to conduct a retrospective and observational single-center study. The selection criteria were: meet the characteristics of IgA dominance or codominance in immunofluorescence, and conform to 3 of the following 5 criteria: 1.Clinical or laboratory evidence show that there is infection before or at the onset of glomerulonephritis; 2.The level of serum complement decreased; 3.Renal pathology is consistent with endocapillary proliferative glomerulonephritis; 4. Glomerular immunofluorescence staining showed complement C3 dominance or codominance; 5. Hump-like subepithelial immune complex deposition was observed under electron microscopy. According to age, sex, renal function (estimated glomerular filtration rate, eGFR) and follow-up time, the control group was constructed with 1:3 matched cases of primary IgA nephropathy. The clinicopathological and prognostic differences between the two groups were analyzed.

**Results:**

The most common histological pattern of IgA-dominant postinfectious glomerulonephritis was acute endocapillary proliferative glomerulonephritis and exudative glomerulonephritis. Immunofluorescence showed mainly IgA deposition or IgA deposition only, mainly deposited in the mesangial area (deposition rate 100 %), with typical C3 high-intensity staining (intensity++~+++), mainly deposited in the mesangial area (deposition rate 92.0 %). The fluorescence intensity of kappa is usually not weaker than lambda. The probability of the appearance of typical hump-like electron deposition under electron microscopy is low. Compared to primary IgA nephropathy, patients with IgA-dominant postinfectious glomerulonephritis had higher proportion of crescents (*p* = 0. 005) and endocapillary hypercellularity (*p* < 0.001) in pathological manifestations. Using serum creatinine level doubled of the baseline or reached end-stage renal disease as the endpoint, the prognosis of IgA-dominant postinfectious glomerulonephritis patients was worse than that of primary IgA nephropathy patients (*p* = 0.013).

**Conclusions:**

The clinicopathological features of patients with IgA-dominant postinfectious glomerulonephritis was different from that of primary IgA nephropathy, and the prognosis was worse.

## Introduction

In 2003, Nasr et al. firstly reported the IgA-dominant postinfectious glomerulonephritis, mostly secondary to skin infections and often related to staphylococcal infection. Diabetes and advanced age were risk factors. The diagnosis mainly depended on renal pathology, which was characterized by diffuse endocapillary proliferative glomerulonephritis under light microscopy, IgA-dominant or codominant immune complex deposits under immunofluorescence and “hump-like” electron-dense deposits in the mesangial area and subepithelium under electron microscopy [1–4]. Reports of the IgA-dominant postinfectious glomerulonephritis have gradually increased in recent years, and many cases have an atypical presentation, making it difficult to differentiate the disease from primary IgA nephropathy, where the prognosis differs considerably. Most of these research were case reports, retrospective reviews or case series in a single center, and the sample size was often small, leading it hard to fully explain the clinicopathological characteristics and prognosis of the disease and the differentiation from similar diseases [5–12]. Therefore, in-depth research of the clinicopathological features and prognosis of IgA-dominant postinfectious glomerulonephritis has an important clinical significance.

In this study, 50 cases of IgA-dominant postinfectious glomerulonephritis were selected from 2009 to 2020 in our hospital, and their clinical and pathological features were analyzed. In addition, 150 patients with primary IgA nephropathy whose age, sex, renal function at diagnosis (estimated glomerular filtration rate, eGFR) and follow-up time matched were selected as a control group to compare the clinical, pathological and prognostic differences, thus providing a reference for clinical diagnosis and treatment.

## Materials and methods

### Patient profiles

Among a total of 6542 patients who underwent renal biopsy in our hospital from January 2009 to October 2020, 50 patients met the selection criteria of IgA-dominant postinfectious glomerulonephritis and were enrolled to conduct a retrospective and observational single-center study. The diagnosis of IgA-dominant postinfectious glomerulonephritis must meet the characteristics of IgA-dominant or codominant IgA in immunofluorescence (IF), and conform to 3 of the following 5 criteria: 1.Clinical or laboratory evidence show that there is infection before or at the onset of glomerulonephritis; 2.The level of serum complement decrease; 3. Renal pathology is consistent with endocapillary proliferative glomerulonephritis; 4. Glomerular immunofluorescence (IF) staining shows complement C3 dominance or codominance; 5. Hump-like subepithelial immune complex deposition is observed under electron microscopy (EM). The patients who met the following situations are excluded: (1) The clinical data are incomplete; (2) There is no definite pathological diagnosis in our hospital. The clinical and pathological data of 50 patients who met the criteria above were collected. In addition, according to age, sex, renal function (estimated glomerular filtration rate, eGFR) and follow-up time, the control group was constructed with 1:3 matched cases of primary IgA nephropathy, and the clinical, pathological and prognostic differences between the two groups were analyzed. The selection criteria of primary IgA nephropathy are as follows: (1) Clinically diagnosed as primary IgA nephropathy; (2) The clinical and pathological data are complete; (3) No other primary or secondary glomerular diseases were found. This study was approved by the Ethics Committee of The First Affiliated Hospital of Wenzhou Medical University.

### Clinical parameters and laboratory data

We collected patients’ basic information, including sex, age, height, weight, blood pressure, etc., and collect relevant laboratory data such as hemoglobin (Hb), serum creatinine (s-Cr), albumin, uric acid, total cholesterol, triglyceride, HDL- cholesterol, LDL- cholesterol, fibrinogen, 24-hour proteinuria and so on. eGFR adopts modified eGFR-EPI formula [10] : Male: eGFR = 144x (s-Cr/0.9)-0.411 × (0.993) ^age^ (s-Cr ≤ 0.9 mg/dl), eGFR = 144x (s-Cr/0.9)-1.209 × (0.993) ^age^ (s-Cr > 0.9 mg/dl). Female: eGFR = 144x (s-Cr/0.7)-0.329 × (0.993) ^age^ (s-Cr ≤ 0.7 mg/dl), eGFR = 144x (s-Cr/0.7)-1.209 × (0.993) ^age^ (s-Cr > 0.7 mg/dl).

### Histopathological examination

All the specimens obtained from renal biopsy were examined by light microscopy (LM) and immunofluorescence (IF). Some of the specimens were examined by electron microscopy (EM). For LM, all the specimens were stained with hematoxylin–eosin (HE), periodic acid-Schiff (PAS), periodic acid methenamine silver (PAM), and elastic-masson trichrome. For IF, frozen sections were stained with the direct immunofluorescent method. Samples for EM were fixed with 2.5 % glutaraldehyde and processed for regular transmission EM. The pathological diagnosis was based on the Oxford pathological classification (MESTC score) criteria, including mesangial hypercellularity(M0/1), endocapillary hypercellularity(E0/1), segmental glomerulosclerosis(S0/S1), tubular atrophy and interstitial fibrosis(T0/1/2), and crescent (C0/1). According to the results of light microscopy, statistics were made on the number of glomeruli, global sclerosis, mesangial hypercellularity, mesangial matrix expansion, endocapillary hypercellularity, crescent, adhesion and so on. According to the results of immunofluorescence, statistics were made on the deposition sites of immunoglobulin(IgG, IgA, IgM), complement(C3, C4, C1q), fibrinogen, light chain (kappa and lambda), and the staining intensity was classified into -、+、++、+++、++++. According to the results of electron microscopy, statistic were made on the mesangial hypercellularity, mesangial matrix hyperplasia, basement membrane thickening, foot process fusion and the sites of electron dense deposition.

### Outcomes

The endpoint was defined as: (1) Serum creatinine increased to (2) 0 times of the baseline, or eGFR decreased by 50 %. 2.Reached end-stage renal disease and received renal replacement therapy; (3) Compound endpoint: serum creatinine level doubled or reached end-stage renal disease; (4) Died. The average follow-up time was 40.16 months (3 months ~ 89 months).

### Statistical analysis

Continuous variables with normal distribution were expressed by mean ± standard deviation, and Student’s t test was used for inter-group comparison. Non-normal continuous variables were expressed by median [quartile], and Wilcoxon rank sum test was used for inter-group comparison. The classification variables were expressed by the number of cases (%), and χ 2 test was used for inter-group comparison. The IgA-dominant postinfectious glomerulonephritis group and primary IgA nephropathy group were matched according to age, sex, eGFR and follow-up time at 1:3, and the propensity score-matching algorithm was used to compare the classification data between the two groups. Cox regression analysis and χ 2 test were used to analyze the risk factors of patients with IgA-dominant post-infectious glomerulonephritis. *p* < 0.05 on a two-tailed test was regarded as statistically significant. The cox regression curve was used to display the endpoint. All data were analyzed and processed by R 4.0.3 software [13].

## Results

### Basic information and medical history of patients

According to the inclusion and exclusion criteria, a total of 50 patients with IgA-dominant postinfectious glomerulonephritis were selected from patients who underwent renal biopsy pathological examination at our centre from January 2009 to October 2020, and their basic information and clinical data were collected, as shown in Table 1. The data were then summarized and the results are shown in Table 2. The average age of the patients at the time of diagnosis was 42.8 years old, the maximum age was 80 years old, and the minimum age was 19 years old. The male-to-female ratio was 1:1.38. Definite pathogens were detected in the course of diagnosis and treatment in 10 patients, including 2 cases of Staphylococcus aureus, 2 cases of Ureaplasma Urealyticum, 2 cases of Escherichia coli, 1 case of Mycobacterium tuberculosis, 1 case of Moraxella catarrhalis, 1 case of Mycoplasma hominis, 1 case of Haemophilus parainfluenzae and 1 case of Streptococcus saliva. There were 31 cases with definite infection site, including 10 cases of urinary tract infection, 9 cases of upper respiratory tract infection, 4 cases of pulmonary infection, 4 cases of skin infection, 3 cases of digestive tract infection and 1 case of abdominal infection. The known time of infection in 10 patients ranged from 1 day to 2 months before treatment, and the exact time of infection in 40 patients was unknown. 20 patients were treated with different antibiotics, and the rest were not treated with antibiotics. In terms of complications, there were 7 cases with viral hepatitis B, 4 cases with diabetes, 4 cases with fatty liver and 2 cases with traumatic fracture.

**Table 1:** IgA-dominant postinfectious glomerulonephritis: demographic and history

Patient	Sex	Age	Pathogen	Infection site	The interval between onset of infections and nephritis	Antibiotics	Complication
1	M	54	S.aureus	Skin	ND	LZD	LC; Foot injury; HTN
2	M	76	S.aureus	Skin	ND	OX	ND
3	M	64	ND	ND	ND	ND	HTN; DM
4	F	57	ND	lung	ND	PRL	NAFLD
5	M	80	ND	Skin	2 months	RL	HTN; Fracture
6	M	62	M.tuberculosis	lung	ND	PRL	HTN; NAFLD
7	F	54	M.catarrhalis	URT	ND	SCF	HTN;
8	F	19	ND	URT	< 2days	CEC; PRL	ND
9	F	34	UU + M.hominis	UT	ND	ND	ND
10	M	49	ND	ND	ND	ND	ND
11	F	27	ND	ND	ND	ND	ND
12	F	46	ND	URT	ND	ND	HTN; Myoma uterus
13	F	31	ND	ND	ND	ND	ND
14	M	58	ND	lung	2weeks	PRL; MEM	HTN; CD; HBV
15	F	74	E.coli	URT	ND	CZO	HTN; DM
16	M	58	ND	ND	ND	ND	HTN
17	F	61	UU	URT	ND	AZM	HTN
18	F	45	ND	ND	ND	ND	HTN
19	F	43	ND	lung	ND	CZO; AZM	HTN; DM
20	M	26	ND	ND	ND	ND	HTN
21	F	19	H.parainfluenzae	URT	ND	ND	ND
22	F	43	ND	ND	ND	ND	HTN; HBV
23	F	27	ND	URT	< 1 day	SCF	ND
24	F	38	ND	ND	ND	ND	Sacroilitis; NAFLD
25	M	36	ND	ND	ND	ND	ND
26	M	20	ND	UT	4days	CRO	ALF
27	F	42	ND	UT	ND	ND	ND
28	M	50	ND	ND	ND	ND	Fracture
29	F	31	ND	ND	ND	ND	ND
30	F	30	ND	URT	1 day	CEC	Acute tonsillitis
31	M	61	ND	ND	ND	ND	HTN
32	F	36	ND	ND	ND	ND	ND
33	F	21	ND	Skin	ND	ND	ND
34	M	27	ND	GIT	< 1 day	ND	ND
35	M	50	ND	ND	ND	ND	ND
36	F	42	ND	UT	ND	LEV	ND
37	F	21	ND	ND	ND	ND	HBV
38	F	70	ND	UT	< 1 day	MEM; SCF	NAFLD
39	F	35	ND	UT	ND	ND	ND
40	F	28	ND	ND	ND	ND	ND
41	M	34	E.coli	UT	ND	LEV	ND
42	F	33	ND	URT	1	ZOX	Bulbar ulcers
43	F	25	ND	URT	< 10days	ND	ND
44	M	38	ND	ND	ND	ND	HTN
45	M	35	ND	URT	ND	ND	HBV
46	M	45	ND	UT	ND	SCF;CEC	HBV
47	M	60	ND	GIT	ND	ND	HTN; HBV
48	M	25	S. salivarius	abdominal	ND	VCA; MXF	HTN
49	F	52	ND	GIT	ND	ND	HBV; Myoma uterus
50	F	49	ND	ND	ND	ND	HTN; DM

Sex: *M* male, *F *female Pathogen: *E.coli* escherichia coli, *H.parainfluenzae* Hemophilus parainfluenzae, *M.catarrhalis* moraxella catarrhalis, *M.hominis* mycoplasma hominis, *M.tuberculosis* mycobacterium tuberculosis, *S.aureus* staphylococcus aureus, *S. salivarius* staphylococcus salivarius, *UU* Ureaplasma Urealyticum

Infection site: *GIT* gastrointestinal tract, *URT* upper respiratory tract, *UT* urinary tract

Antibiotics: *AZM* azithromycin, *CEC* cefaclor, *CRO* ceftriaxone, *CZO* cefazolin, *LEV* levofloxacin, *LZD* linezolid, *MEM* meropenem, *MXF* moxifloxacin, *OX* oxacillin, *PRL* piperacillin, *RL* Sulfamethoxazole, *SCF* Cefperazone sulbactam, *VCA* vancomycin, *ZOX* cefuroxime

Complications: *ALF* abnormal liver function, *CD* crohn disease, *DM* diabetes mellitus, *LC* live cirrhosis, *HTN* hypertesion, *HBV* hepatitis B, *NAFLD* non-alcoholic fatty liver disease, *ND* no data

**Table 2:** IgA-dominant postinfectious glomerulonephritis: summary of demographic and history

	Total (N = 50)
Sex	
F	29 (58.0 %)
M	21 (42.0 %)
Pathogen	
E.coli	2 (4.0 %)
H.Parainfluenzae	1 (2.0 %)
M.catarrhalis	1 (2.0 %)
M.Tuberculosis	1 (2.0 %)
UU	2 (4.0 %)
M.Hominis	1 (2.0 %)
S.Aureus	2 (4.0 %)
S.Salivarius	1 (2.0 %)
ND	40 (80.0 %)
Infection site	
Abdominal	1 (2.0 %)
GIT	3 (6.0 %)
Lung	4 (8.0 %)
Skin	4 (8.0 %)
URT	9 (18.0 %)
UT	10 (20.0 %)
ND	19 (38.0 %)
The interval of onset of infections and nephritis	
<3days	6 (12.0 %)
3days-2weeks	3(6.0 %)
>2weeks	1 (2.0 %)
ND	40 (80.0 %)
Complication	
Acute Tonsillitis	1 (2.0 %)
ALF	1 (2.0 %)
Bulbar Ulcers	1 (2.0 %)
CD	1 (2.0 %)
DM	4 (8.0 %)
Foot Injury	1 (2.0 %)
Fracture	2 (4.0 %)
HBV	7 (14.0 %)
HTN	18 (36.0 %)
LC	1 (2.0 %)
Myoma Uterus	2 (4.0 %)
NAFLD	4 (8.0 %)
Sacroilitis	1 (2.0 %)
ND	20 (40.0 %)

Sex: *M* male, *F* female

Pathogen: *E.coli* escherichia coli, *H.parainfluenzae* Hemophilus parainfluenzae, *M.catarrhalis* moraxella catarrhalis, *M.hominis* mycoplasma hominis, *M.tuberculosis* mycobacterium tuberculosis, *S.aureus* staphylococcus aureus, *S. salivarius* staphylococcus salivarius, *UU* Ureaplasma Urealyticum

Infection site: *GIT* gastrointestinal tract, *URT* upper respiratory tract, *UT* urinary tract

Complications: *ALF* abnormal liver function, *CD* crohn disease, *DM* diabetes mellitus, *LC* live cirrhosis, *HTN* hypertesion,*HBV* hepatitis B, *NAFLD* non-alcoholic fatty liver disease, *ND* no data

### Renal pathological manifestations of IgA-dominant postinfectious glomerulonephritis

#### Light microscopy

**Fig. 1:**
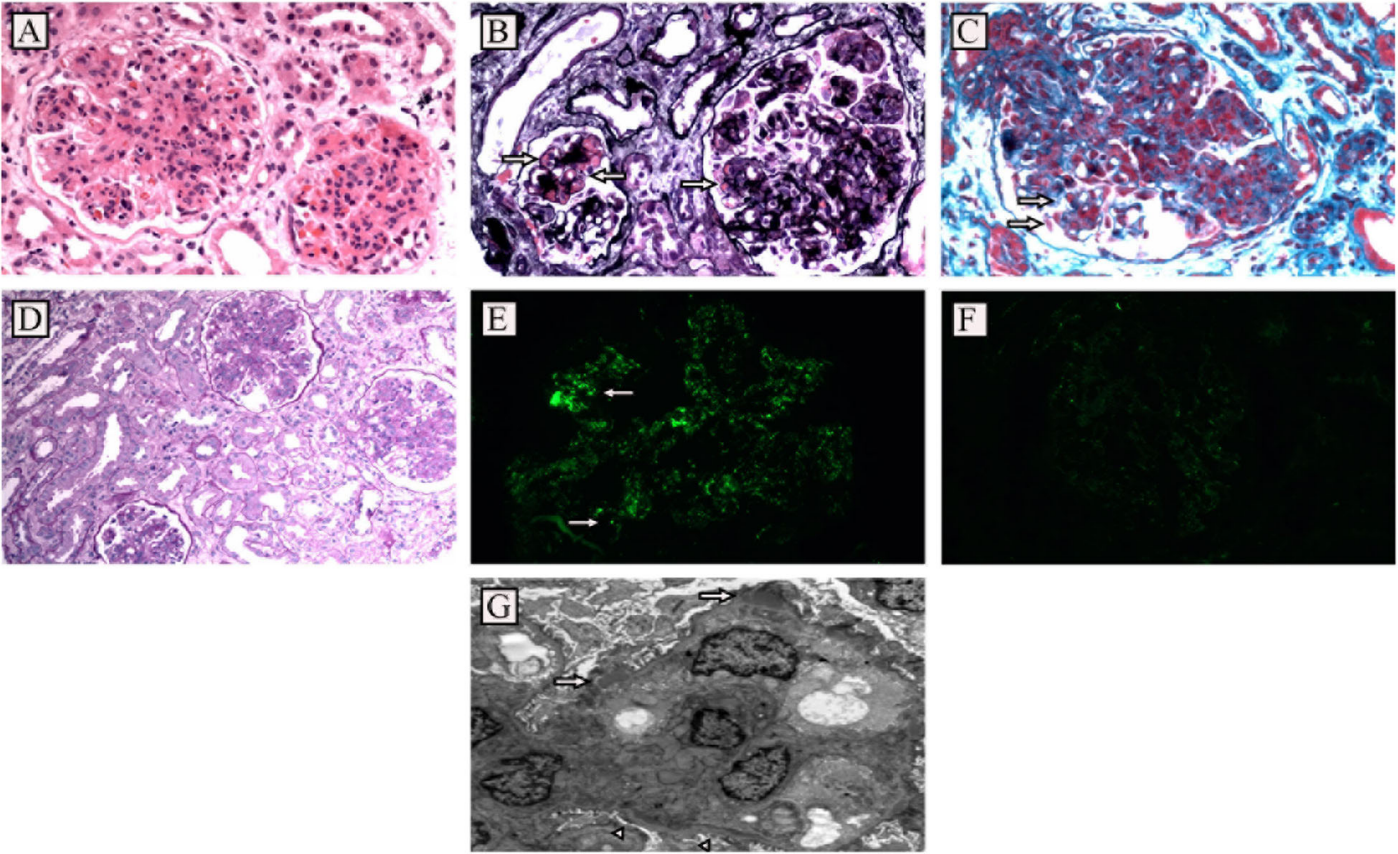
**A**: Light microscopy findings in renal biopsy: There is marked global endocapillary hypercellularity with numerous infiltrating neutrophils causing obliteration of the capillary lumina. (hematoxylin and eosin: ×200). **B**: Light microscopy findings in renal biopsy: There is marked global endocapillary hypercellularity and subendothelial deposits (arrow) ,mesangial deposits.(methenamine silver: ×200) **C**: Light microscopy findings in renal biopsy: There is marked global endocapillary hypercellularity and mesangial, “hump”-like deposits (arrow) (Masson’s trichrome: ×200) **D**: Light microscopy findings in renal biopsy: All glomeruli show diffuse endocapillary hypercellularity and neutrophilic infiltration, tubulointerstitial changes are mild. (periodic acid-Schiff: ×200 ) **E**: C3 immunostaining. There is coarsely granular glomerular staining: globally in the mesangium and segmentally in the glomerular capillary walls in the distribution of subepithelial humps ( IF: ×400). **F**: IgA immunostaining. There is bright, coarsely granular glomerular staining: globally in the mesangium and segmentally in the glomerular capillary walls in the distribution of subepithelial humps (arrows; IF:×400). **G**: Transmission electron microscopy showing subepithelial deposits (arrows), the upper deposits with hump characteristics. Also shown are scattered mesangial deposits (arrow heads) ×2000

The results are shown in Table 3. In this study, the average number of glomeruli in renal biopsies was 22.18. The most common histological pattern of IgA-dominant postinfectious glomerulonephritis is acute endocapillary proliferative glomerulonephritis and exudative glomerulonephritis, similar to acute post-streptococcal glomerulonephritis, showing acute diffuse endocapillary proliferative glomerulonephritis with significant neutrophil and monocyte endocapillary infiltration and obvious lumen blockage (Fig. 1A-D). This pattern accounted for 80.0 % of the cases. Other histological patterns included mesangial proliferative glomerulonephritis, membranous proliferative glomerulonephritis and crescentic glomerulonephritis, accounting for 12.0 %, 6.0 and 2.0 % of the cases, respectively. Mesangial hypercellularity occurred in 94.0 % of patients, and mesangial matrix expansion occurred in 84.0 % of patients. The average number of global sclerosis is 3.33. Adhesion occurred in 50.0 % of patients. Crescent appeared in 38 cases, mostly small crescent, including 27 cases of cellular crescent, 26 cases of fibrocelluar crescent and 26 cases of fibrous crescent. At the same time, there were 3 cases of hypertensive nephropathy and 1 case of diabetic nephropathy. Most patients had renal interstitial inflammation, showing interstitial lymphocyte and plasma cell infiltration, renal tubular atrophy and renal interstitial fibrosis. 12 cases accompanied with acute interstitial inflammation, including 8 cases of mild inflammation, 3 cases of medium inflammation and 1 case of heavy inflammation. 20 cases accompanied with chronic interstitial inflammation, including 5 cases of mild inflammation, 14 cases of medium inflammation and 1 case of heavy inflammation. Most patients had varying degrees of vascular lesions, including mild endarterium hyperplasia in 16 cases, medium endarterium hyperplasia in 12 cases, heavy endarterium hyperplasia in 3 cases, and hyalinosis in 28 cases.

**Table 3:** IgA-dominant postinfectious glomerulonephritis: Light microscopy

	Statistical result
	Total = 50
Number of glomeruli(mean)	22.2
Global sclerosis (mean)	3.3
Histological pattern	
Acute endocapillary proliferative glomerulonephritis(%)	40(80 %)
Mesangial proliferative glomerulonephritis(%)	6(12.0 %)
Membrane proliferative glomerulonephritis(%)	3(6.0 %)
Crescent glomerulonephritis(%)	1(2.0 %)
Mesangial hypercellularity(%)	47(94.0 %)
Mesangial matrix expansion(%)	42(84.0 %)
Endocapillary hypercellularity (%)	40(80 %)
Adhesion(%)	25(50.0 %)
Cases with crescent	
Cellular crescent(%)	27(54.0 %)
Fibrocellular crescent(%)	26(52.0 %)
Fibrous crescent(%)	26(52.0 %)
Interstitial inflammation change	
Acute-mild inflammation(%)	8(16.0 %)
Acute-medium inflammation(%)	3(6.0 %)
Acute-heavy inflammation(%)	1(2.0 %)
Chronic-mild inflammation(%)	5(10.0 %)
Chronic-medium inflammation(%)	14(28.0 %)
Chronic-heavy inflammation(%)	1(2.0 %)
Vasculopathy	
Mild-endarterium hyperplasia(%)	16(32.0 %)
Medium-endarterium hyperplasia(%)	12(24.0 %)
Hard-endarterium hyperplasia(%)	3(6.0 %)
Hyalinosis(%)	28(56.0 %)

#### Immunofluorescence

The results are shown in Table 4. The fluorescent tissue contains an average of 4.2 glomeruli (2 ~ 9 glomeruli). IgA is the dominant or co-dominant immunoglobulin deposited in glomeruli (intensity +~++++). The mode of deposition is coarse granular or massive deposition in glomerular mesangial area and / or capillary wall. The deposition rate of IgA was 100 % in the mesangial area and 18.0 % in the capillary wall. Typical C3 high-intensity staining (intensity ++~+++)was observed. 92 % of the cases showed C3 deposited in the mesangial area and 22.0 % in the capillary wall. The deposition rates of IgG and IgM in the mesangial area and capillary wall were 22.0 %、6.0 and 42.0 %、16.0 %, respectively. C1q and C4 staining were negative in most patients. In addition, fibrinogen deposition was found in 10.0 % of the patients in the capillary wall and 20.0 % of the patients in the mesangial area. 32 cases were positive for kappa staining (intensity+~+++), and 42 cases were positive for lambda staining(intensity+~+++). The staining intensity of kappa was usually not weaker than that of lambda (Fig. 1E, F), which was different from primary IgA nephropathy ( In primary IgA nephropathy, the staining intensity of lambda is usually not weaker than that of kappa).

**Table 4:** IgA-dominant postinfectious glomerulonephritis: Immunofluorescence and Electron microscopy

	Statistical result
Immunofluorescence	Total = 50
IgA Capillary (%)	9(18.0 %)
IgA Mesangium(%)	50(100 %)
IgG Capillary (%)	3(6.0 %)
IgG Mesangium(%)	11(22.0 %)
IgM Capillary (%)	8(16.0 %)
IgM Mesangium(%)	21(42.0 %)
C1q Capillary (%)	1(2.0 %)
C1q Mesangium(%)	0(0.0 %)
C3 Capillary (%)	11(22.0 %)
C3 Mesangium(%)	46(92 %)
C4 Capillary (%)	1(2.0 %)
C4 Mesangium(%)	1(2.0 %)
Fibrinogen Capillary (%)	5(10.0 %)
Fibrinogen Mesangium(%)	10(20.0 %)
Kappa(%)	32(64.0 %)
Lambda(%)	42(84.0 %)
Electron microscopy	Total = 14
Basement membrane thickening(%)	0(0.0 %)
Endotheliocytosis (%)	11(78.5 %)
Foot process fusion (%)	14(100 %)
Mesangial hypercellularity(%)	10(71.4 %)
Mesangial matrix expansion (%)	9(64.3 %)
Dense deposits	
Mesangium (%)	10(71.4 %)
Paramesangium(%)	7(50.0 %)
Subepithelial (%)	3(21.4 %)
Subendothelial(%)	1(7.1 %)
Intramembrane(%)	0(0.0 %)
Hump (%)	1(7.1 %)

#### Electron microscopy

The results are shown in Table 5. 14 of the 50 IgA-dominant postinfectious glomerulonephritis cases were examined by electron microscopy. Ultrastructurally, 71.4 % of the cases showed electronic dense deposits in the mesangial area, 50 % in the paramesangial area, 21.4 % in the subepithelial and 7.1 % in the subendothelial. Only one patient showed hump-like deposits under the electron microscopy, accounting for 7.1 % (Fig. 1G). Foot process fusion occurred in all patients.

**Table 5:** Pathological prifiles under electron microscopy in this study

	Statistical result
Microscopy(Electron microscopy, *n* = 14)	
Basement membrane thickening(%)	0(0.0 %)
Endotheliocytosis (%)	11(78.5 %)
Foot process fusion (%)	14(100 %)
Mesangial hypercellularity(%)	10(71.4 %)
Mesangial matrix expansion (%)	9(64.3 %)
Dense deposits	
Mesangium (%)	10(71.4 %)
Paramesangium(%)	7(50.0 %)
Subepithelial (%)	3(21.4 %)
Subendothelial(%)	1(7.1 %)
Intramembrane(%)	0(0.0 %)
Hump (%)	1(7.1 %)

### Comparison of clinical data between IgA-dominant postinfectious glomerulonephritis and primary IgA nephropathy

After matching age, sex, eGFR and follow-up time, there was no significant difference in demographic data between IgA-dominant postinfectious glomerulonephritis group (right column of Table 6) and primary IgA nephropathy group (left column of Table 6). As shown in Table 6, IgA-dominant postinfectious glomerulonephritis group had higher average serum total cholesterol level (*p* < 0.001), higher average serum high density lipoprotein level (*p* = 0.039), lower average serum low density lipoprotein level (*p* = 0.021), higher average 24-hour urinary protein level (*p* < 0.004), lower average serum albumin level (*p* < 0.001), higher average fibrinogen level (*p* = 0. 001), higher proportion of crescent (*p* = 0. 005), and higher proportion of endocapillary hypercellularity (*p* < 0.001). In terms of treatment, patients with IgA-dominant postinfectious glomerulonephritis got more aggressive treatment, using more steroids (*p* = 0.005) and less ACEI (*p* = 0.03).

**Table 6:** Comparision between IgA-DPIGN group and IgAN group

	IgAN	IgA-DPIGN	*p*-value
n	150	50	
female	90	29	
Age (year, mean (SD))	43.58 (13.40)	42.82 (16.05)	0.742
Systolic pressure(mmHg,mean (SD))	132.20 (20.03)	130.75 (19.63)	0.671
Diastolic pressure (mmHg,mean (SD))	79.82 (11.07)	76.56 (10.73)	0.085
BMI (mean (SD))	23.26 (3.17)	23.58 (4.24)	0.603
MBP (mmHg,mean (SD))	97.28 (12.42)	94.62 (12.43)	0.213
Follow-up month (month,mean (SD))	24.53 (26.24)	24.80 (25.35)	0.95
Total cholesterol (mmol/L,mean (SD))	4.80 (1.14)	5.50 (1.68)	0.001
Triglyceride (mmol/L,mean (SD))	1.86 (1.00)	1.66 (0.89)	0.209
HDL(mmol/L,mean (SD))	1.08 (0.29)	1.19 (0.41)	0.039
LDL(mmol/L,mean (SD))	2.82 (0.90)	3.22 (1.38)	0.021
Serum creatinine (mg/dL,mean (SD))	1.44 (1.65)	1.39 (1.07)	0.837
Proteinuria(g/d,mean(SD))	1.96 (2.38)	3.24 (3.41)	0.004
Serum albumin(g/L,mean(SD))	37.78 (7.38)	32.28 (6.48)	< 0.001
Uric acid(mg/dL,mean (SD))	5.69 (1.78)	5.97 (1.64)	0.056
Hemoglobin (g/L,mean(SD))	119.73 (19.10)	116.45 (16.65)	0.283
Fibrinogen(g/L,mean(SD))	3.55 (1.04)	4.19 (1.37)	0.001
eGFR(mL/(min•1.73 m^2^ )mean(SD))	76.14 (34.75)	73.87 (34.16)	0.688
Pathological report			
M1 (%)	63 (42.3)	25 (50.0)	0.432
E1 (%)	34 (22.8)	39 (78.0)	< 0.001
S1 (%)	109 (73.2)	33 (66.0)	0.431
Tscore (%)			0.216
T0	80 (53.7)	21 (42.0)	
T1	38 (25.5)	19 (38.0)	
T2	31 (20.8)	10 (20.0)	
C1 (%)	57(38.0)	29 (58.0)	0.005
Treatment			
ACEI (%)	22 (14.7)	1 (2.0)	0.03
ARB (%)	79 (52.7)	22 (44.0)	0.369
Steroid (%)	46 (30.7)	27 (54.0)	0.005
CTX (%)	10 (6.7)	6 (12.0)	0.367
otherIM (%)	13 (8.7)	1 (2.0)	0.201

*BMI *body mass index, *MBP *mean blood pressure, *HDL *high density lipoprotein, *LDL *low density lipoprotein, *eGFR *estimated glomerular filtrationrate, *MESTC-score* the assessment of mesangial hypercellularity/endocapillary hypercelluarrity/segmental sclerosis/renal tubular lesion/crescent according to the Oxford classification, *M1* mesangial hypercellularity, *E1* endocapillary hypercellularity, *S1* segmental glomerulosclerosis, *T1/2* tubular atrophy and interstitial fibrosis > 25 %, *C1* crescents in at least one glomerulus, *ARB *angiotensin receptor antagonist, *IM * immunosuppressant, *CTX *cyclophosphamide, *ACEI *angiotesin-converting enzyme, *IgA-DPIGN *IgA-dominant postinfectious glomerulonephritis, *IgAN *primary IgA nephropathy

All the data above are acquired before the renal biopsy

### Prognosis and risk factors

**Fig. 2:**
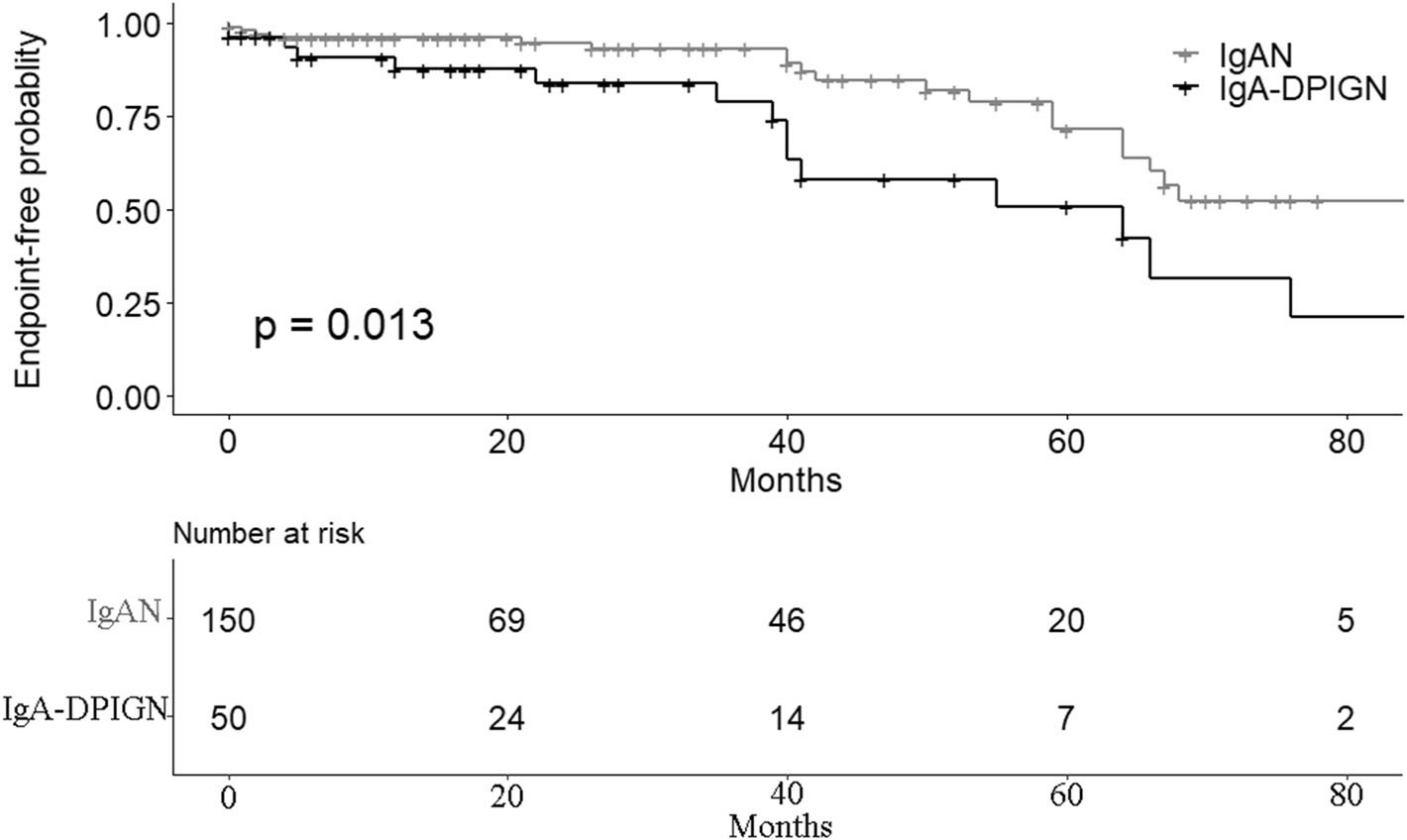
Comparison of prognosis between IgA-DPIGN group and IgAN group (Kaplan-Meier curve), *p*-value = 0.0013. The endpoint is that the serum creatinine level doubled of the baseline or reached end stage renal disease. IgA-DPIGN, IgA-dominant postinfectious glomerulonephritis; IgAN, primary IgA nephropathy

The average follow-up time in this study was 40.16 months. When regarding serum creatinine doubling or reaching end-stage renal disease as the compound endpoint, the prognosis of patients with IgA-dominant postinfectious glomerulonephritis was worse than that of primary IgA glomerulonephritis (*p* = 0.013), as shown in Fig. 2. Using serum creatinine doubling or entering end-stage renal disease as the compound endpoint, univariate cox regression analysis was performed on the related factors of IgA-dominant postinfectious glomerulonephritis. As shown in Table 7, factors that have statistical differences(*p* < 0.1) were sent to make the selection(Forward-backward stepwise regression method) according to the Akaike information criterion and the factors remained were selected to make multivariate analysis. The results showed that the increase of serum albumin level was an independent protective factor (*p* < 0.004), and the increased serum creatinine level (*p* < 0.001), serum uric acid level (*p* = 0.01) and decreased eGFR level (*p* < 0.001) were independent risk factors. Renal tubular atrophy or interstitial fibrosis > 50 % (*p* = 0.20) and the existence of crescents (*p* = 0.19) were risk factors, but there was no significant statistical difference, as shown in Table 7.

**Table 7:** Cox regression analysis

	Univariate analysis		Multivariate analysis	
Factors	HR(95 %CI for HR)	*p*-value	HR(95 %CI for HR)	*p*-value
Age (year)	0.99 (0.96-1.00)	0.27		
Female	0.42 (0.21–0.81)	0.01		
Proteinuria (g/24-h)	1.20 (1.10–1.40)	< 0.001		
Total cholesterol (mmol/L)	1.40 (1.10–1.70)	0.009		
Triglyceride (mmol/L)	1.20 (0.84–1.70)	0.31		
High density lipoprotein (mmol/L)	0.83 (0.25–2.80)	0.76		
Low density lipoprotein (mmol/L)	1.70 (1.30–2.20)	< 0.001		
Serum creatinine (mg/dL)	1.60 (1.40–1.90)	< 0.001	1.99(1.56–2.55)	< 0.001
Serum albumin (g/L)	0.91 (0.86–0.96)	0.001	0.92(0.86–0.97)	0.004
Serum uric acid (mg/dL)	1.30 (1.10–1.60)	0.002	1.33(1.07–1.65)	0.01
Hemoglobin (g/L)	0.99 (0.97-1.00)	0.64		
Fibrinogen (g/L)	1.30 (1.10–1.60)	0.015		
M1	1.60 (0.80–3.20)	0.19		
E1	2.00 (1.00-3.80)	0.044		
S1	1.50 (0.63–3.60)	0.36		
T score				
T0	refer	-	refer	-
T1	1.20 (0.63–2.40)	0.54		
T2	3.20 (1.60–6.50)	0.002	2.02(0.69–5.95)	0.20
C1	2.30 (1.20–4.50)	0.016	1.65(0.79–3.48)	0.19
IgA-DPIGN	2.30 (1.20–4.40)	0.016	3.98(1.69–9.37)	0.002
eGFR (mL/(min•1.73 m^2^ ))	0.99 (0.98-1.00)	0.01	1.02(1.01–1.04)	< 0.001

Endpoint is the increase of serum creatinine to 2 times the base line or end-stage renal diseasse. Factors that have statistical differences (*p* < 0.1) were sent to make the selection(Forward-backward stepwise regression method) according to the Akaike information criterion and the factors remained were selected to make multivariate analysis

*HR* Hazard ratio, *CI* confidence interval, *eGFR* estimated glomerular filtration rate, *MESTC-score* the assessment of mesangial hypercellularity/endocapillary hypercelluarrity/segmental sclerosis/renal tubular lesion/crescent according to the Oxford classification, *M1* mesangial hypercellularity, *E1* endocapillary hypercellularity, *S1* segmental glomerulosclerosis, *T1/2 *tubular atrophy and interstitial fibrosis > 25 %, *C1/2* crescents in at least one glomerulus, *IgA-DPIGN* IgA-dominant postinfectious glomerulonephritis

All the data above are acquired before the biopsy

## Discussion

IgA-dominant postinfectious glomerulonephritis, as a special form of postinfectious glomerulonephritis, has been reported increasingly since it was first proposed by Nasr et al. in 2003. Most of the classic cases are secondary to skin infection and often relate to staphylococcal infection. Diabetes and advanced age are the risk factors. The diagnosis mainly depends on renal pathology, which is characterized by diffuse endocapillary proliferative glomerulonephritis under light microscopy, IgA-dominant or codominant immune complex deposits under immunofluorescence, and hump-like electron dense deposition in mesangial area and subepithelium under electron microscopy [1, 14]. However, there were also reports of atypical cases of IgA-dominant postinfectious glomerulonephritis, which were difficult to be distinguished from other diseases. For example, Mark Haas et al. [4] and Yao-Ko Wen et al. [15] reported 13 cases and 10 cases of IgA-dominant postinfectious glomerulonephritis respectively. They pointed out that IgA-dominant postinfectious glomerulonephritis was similar to post-streptococcal glomerulonephritis in light microscopy, immunofluorescence and ultrastructure. The underlying infection may be subclinical, non-staphylococcal or both, and can occur in both diabetic and non-diabetic patients. Eric Wallace et al. [3] reported a case of IgA-dominant postinfectious glomerulonephritis associated with colitis caused by Clostridium difficile. Its pathogenesis was similar to that of active IgA nephropathy, and the clinical and pathological manifestations were highly similar. Marc Saad et al. [8]also reported a case with pulmonary-renal syndrome as the main manifestation. The above study found some difficulty in differentiating IgA-dominant postinfectious glomerulonephritis when the presentation is atypical from other glomerulonephritis, especially primary IgA nephropathy, which can have similar light microscopy and immunofluorescence manifestations, but the treatment options for the two are different, and the prognosis of patients with IgA-dominant postinfectious glomerulonephritis is often reported worse [15–17]. However, the above studies are mainly case reports, or retrospective reviews, or single-center case series, and the sample size is often small, so it is difficult to fully explain the clinicopathological characteristics, prognosis and differentiation of the disease from other similar diseases.

In our study, the selection criteria of IgA-dominant postinfectious glomerulonephritis were established with reference to the criteria of Handa, Nasr and others. A total of 6542 cases of renal biopsy were performed in our center from 2009 to 2020. We selected 50 cases of IgA-dominant postinfectious glomerulonephritis which were clinically and pathologically diagnosed, and studied their clinical and pathological features. In addition, 150 patients with primary IgA nephropathy matched with age, sex, eGFR and follow-up time were selected and matched according to 1:3, and the differences in clinical, pathology and prognosis were analyzed. The results are as follows.

### Infection site and pathogen

It has been previously reported that classic IgA-dominant postinfectious glomerulonephritis is often secondary to skin infection, and mostly related to staphylococcal infection [1]. However, our study found that upper respiratory tract and urinary tract infections were more often than skin infections. The detectable rate of staphylococci was not high (4 %), and atypical pathogens such as Escherichia coli and Ureaplasma Urealyticum could be detected in a few patients. Most pathogens could not be found (pathogen detectable rate was 20 %). The proportion of patients with Hepatitis B is relatively high (14 %). The reason may be due to the high prevalence rate of Hepatitis B in China, or there may be some correlation between the two diseases which was mentioned by Miquelestorena-Standley, E. et al. in their report and is worthy of further study [12].

### Laboratory examination

Compared with primary IgA nephropathy, IgA-dominant postinfectious glomerulonephritis had more proteinuria and lower serum albumin level, indicating that it had greater damage on glomerular filtration barrier, which may be one of the reasons for more serious disease progression. On the other hand, patients with IgA-dominant postinfectious glomerulonephritis had higher serum fibrinogen levels. As an inflammatory factor, fibrinogen suggests that the inflammation in this disease may be more severe, or it may be used as a clinical feature for differentiation from primary IgA nephropathy.

### Renal pathological changes

The pathological manifestations of IgA-dominant postinfectious glomerulonephritis have been reported in the past, mainly were endocapillary proliferative glomerulonephritis under light microscopy, IgA-dominant or codominant immune complex deposits under immunofluorescence, and hump-like dense depositions in the mesangial area and subepithelium under electron microscopy [6, 14]. Our findings are alomst consistent with the above. Under light microscopy, compared with primary IgA nephropathy, IgA-dominant postinfectious glomerulonephritis was more likely to form crescents (*p* = 0.05), and endocapillary hypercellularity were more severe (*p* < 0.001), accompanied by significant neutrophil and monocyte capillary infiltration, and obvious blockage of the lumen, indicating that the disease may have more serious effect and destruction on renal structure. The manifestation of immunofluorescence was very similar to that of IgA nephropathy, which showed IgA dominant or co-dominant deposition in the mesangial area. The main differentiation may depend on the comparison of fluorescence staining intensity of lambda and kappa. In IgA-dominant postinfectious glomerulonephritis, the fluorescence staining intensity of kappa is often not weaker than that of lambda, while the immunofluorescence of primary IgA nephropathy often showed the opposite, which is consistent with our findings. Under electron microscopy, hump-like deposits are considered to be the typical manifestation [2], but our study found that the probability of typical hump-like deposits is low (only 1 out of 14 cases found typical hump-like deposits). Therefore, it may be not feasible to rely on the results of electron microscopy as differential diagnosis. The retrospective comparative study of TakayaHanda et al. [3] showed that IgA-dominant postinfectious glomerulonephritis was characterized by the immunoglobulin and complement deposition along the glomerular capillary wall accounted for 15.4-54 %, which was different from primary IgA nephropathy. Miquelestorena-Standley, E. et al. reported that IgA depositsion along capillary wall accounted for 19.2-46.2 % [12]. However, our study found that immunoglobulins and complements mainly deposited in the mesangial area (0.0-100 %), and capillary wall deposition only accounted for 2.0-22.0 %.

### Prognosis and its influencing factors

Our study found that in patients with IgA-dominant postinfectious glomerulonephritis, the higher level of serum creatinine and uric acid at diagnosis led to the worse prognosis, which is consistent with previous studies, suggesting that patients with severe renal function damage and elevated uric acid should be treated more actively. The pathological results showed that renal tubulointerstitial atrophy and sclerosis > 50 %(T2) and crescent(C1) were also risk factors for poor prognosis, but there was no statistical difference. Since Bogdan Obrişcă et al. [18] have argued that whether MESTC score can be routinely applied to patients with secondary IgAN remained unsure, the result may need more in-depth studies. Previous studies have suggested that sex and age are risk factors for the disease, but our investigation found that age and sex may be confounding factors and were not directly related to the prognosis of the disease.

Previous studies showed that IgA-dominant postinfectious glomerulonephritis had a poorer prognosis comparing to primary IgA nephropathy. For example, the retrospective comparative study of Takaya Handa et al. [6] found that the prognosis of IgA-dominant postinfectious glomerulonephritis was worse than that of primary IgA nephropathy, which may be due to the fact that the former has more obvious systemic inflammation and local inflammation of glomerular capillary wall. After an average follow-up of 40.16 months, our study found that the prognosis of patients with IgA-dominant postinfectious glomerulonephritis was worse than that of primary IgA glomerulonephritis, and was not disturbed by other factors, which was consistent with the previous studies.

Some of the results of our study were different from previous studies, indicating that there may be some limitations in the previous understanding of IgA-dominant postinfectious glomerulonephritis. The so-called “typical manifestations” may cover only the minority [19–21], and there may be diversity in pathogens, inducements, clinical, pathology and prognosis [22].However, there are still some disadvantages in our study, such as small sample size, few specimens examined by electron microscopy, relatively short time of follow-up, and was only a retrospective and observational single-center study, so the clinicopathological and prognostic characteristics of IgA-dominant postinfectious glomerulonephritis need to be further studied by multicenter with larger samples.

## Conclusions

The pathology and clinical manifestations of IgA-dominant postinfectious glomerulonephritis have certain characteristics, and the differential diagnosis from other similar diseases depends on clinical and pathological features. The prognosis of IgA-dominant postinfectious glomerulonephritis is worse than that of primary IgA nephropathy, which may be related to stronger inflammatory reaction caused by infection-mediated IgA deposition, but its clinicopathological and prognostic characteristics need to be further studied.

## Data Availability

The datasets used and/or analysed during the current study are available from the corresponding author on reasonable request.
